# Intraoperative slow-release dexamethasone intravitreal implant (Ozurdex) in epiretinal membrane peeling surgery: a prospective randomized controlled trial

**DOI:** 10.3389/fphar.2023.1219861

**Published:** 2023-09-01

**Authors:** Siying Li, Qiaozhu Zeng, Li Zhu, Wenbo Liu, Yujing Li, Jiarui Li, Xiaoxin Li, Mingwei Zhao, Jinfeng Qu

**Affiliations:** Department of Ophthalmology, Peking University People’s Hospital, Eye Diseases and Optometry Institute, Beijing Key Laboratory of Diagnosis and Therapy of Retinal and Choroid Diseases, College of Optometry, Peking University Health Science Center, Beijing, China

**Keywords:** epiretinal membrane, pars plana vitrectomy, Ozurdex, central retinal thickness, best-corrected visual acuity

## Abstract

**Purpose**: This study aimed to determine the efficacy of the dexamethasone (DEX) intravitreal implant for the regression of macular edema and the improvement of best-corrected visual acuity (BCVA) after the removal of idiopathic epiretinal membrane (ERM).

**Methods**: This prospective randomized controlled trial recruited 81 patients with idiopathic ERM. These patients all underwent 25-gauge pars plana vitrectomy combined with ERM and internal limiting membrane peeling surgery. Among them, 41 eyes in the DEX group received additional DEX implants and 40 in the non-DEX group did not. Outcomes including central retinal thickness (CRT), BCVA, and intraocular pressure were measured 1 and 3 months after surgery.

**Results**: The DEX group had thinner CRTs compared to the non-DEX group at 1 month postoperatively (*p* <0.05), but did not differ significantly at the 1-week and 3-month follow-up visits (*p* = 0.109 and *p* = 0.417, respectively). There were no statistical differences with respect to BCVA (*p* = 0.499, 0.309, 0.246, and 0.517, respectively) and intraocular pressure (*p* = 0.556, 0.639, 0.741, and 0.517, respectively) between the two groups at each point of follow-up visits.

**Conclusion**: DEX accelerated the reduction of CRT at 1 month after surgery. However, no evidence of further anatomical (CRT) or functional (BCVA) benefits using DEX was observed at 3 months.

**Clinical Trial Registration:** https://clinicaltrials.gov/, identifier NCT05416827.

## Introduction

Idiopathic epiretinal membrane (ERM) refers to a non-vascularized fibrocellular contractile proliferation at the vitreoretinal interface in the macular area, which comprises Müller cells, fibrous astrocytes, and accessory retinal glial cells. It is capable of leading to retinal edema-accompanied macular distortion, underlying blood–retinal barrier breakdown, and eventually, central vision loss and metamorphopsia (Cheung et al., 2017). The prevalence of the ERM ranges from 4% to 12.8%, in accordance with the study population. Bilateral involvement occurs in nearly 10%–20% of cases, generally subjected to asymmetry (Bu et al., 2014; Fung et al., 2021). Pars plana vitrectomy (PPV) with membrane peeling has been extensively demonstrated for symptomatic ERMs; however, macular edema regression and visual acuity increase are often slow (Reilly et al., 2015). Postsurgical macular edema can present in nearly 25%–27% of ERM cases with or without combined cataract extraction, and this incidence can be even higher in the higher phase of ERMs (Iuliano et al., 2021). Several recent publications have proposed that triamcinolone acetonide (TA), intravitreally injected when the surgery is nearly completed, was capable of accelerating intraretinal edema resorption and hastening visual acuity enhancement (Asahi et al., 2020; Mandelcorn et al., 2022). Nevertheless, its application has been limited by the increase of intraocular pressure (IOP) and the risk of postoperative uveitis mimicking infectious endophthalmitis.

Dexamethasone refers to a potent synthetic member that pertains to steroid drugs’ glucocorticoid class, exhibiting anti-inflammatory and immunosuppressant activity 6 times better than triamcinolone and 30 times better than cortisol (Keck et al., 2003). A slow-release dexamethasone (DEX) implant (Ozurdex; Allergan, Inc., Irvine, CA) can deliver dexamethasone for 3–4 months after a single injection, even in vitrectomized eyes. Since cystoid macular edema generally peaks at 4–6 weeks after the completion of the surgery, the use of Ozurdex may provide a more stable and long-term effect to expedite the regression of retinal edema (Boyer et al., 2011; Medeiros et al., 2014). Related research studies are rarely found (Savastano et al., 2021). Accordingly, a prospective randomized controlled trial was designed to determine the dexamethasone (Ozurdex) intravitreal implant’s efficacy to expedite macular edema regression and the enhancement of best-corrected visual acuity (BCVA) after the removal of idiopathic ERM.

## Subjects and methods

### Design

This study was a prospective, single-center, randomized controlled clinical trial.

### Patients

Idiopathic ERM patients with vitrectomy indication at Peking University People’s Hospital between January 2022 and August 2022 were eligible. The inclusion criteria were as follows: (a) patients aged 50 years or above with idiopathic ERMs; (b) symptomatic patients with Snellen BCVA <20/30 and 20/200; (c) central retinal thickness (CRT) >300 μm; (d) ocular axial length less than 26.00 mm; and (e) phakic eyes or pseudophakic eyes with an intact posterior capsule.

The exclusion criteria included the following: (a) concomitant or previous macular diseases that may hinder visual improvement other than ERM (e.g., age-associated macular degeneration, retinal vein occlusion, or diabetic macular edema); (b) previous vitreoretinal surgery or intravitreal injection history; (c) ERMs with lamellar or full-thickness macular holes; (d) eyes that require tamponade or additional treatment (e.g., laser photocoagulation) during follow-up periods; (e) patients with uncontrolled systemic diseases or infectious diseases; and (f) patients who took medicines that may have ocular side effects, such as glucocorticoid or hydroxychloroquine.

The cases were randomized at a ratio of 1:1 as the non-DEX and DEX groups. A computerized randomization table was used to generate the randomization sequence.

### Baseline and follow-up visit

We recorded the baseline demographics and systematic conditions (e.g., hypertension and diabetes mellitus (DM)). All cases received a comprehensive ophthalmic examination, which comprised a refraction test, spectral-domain optical coherence tomography (SD-OCT) (CIRRUS HD-OCT Model 5000, Carl Zeiss Meditec, Germany), color fundus photography, indirect ophthalmoscopy, slit lamp examination, IOP, BCVA, ultra-widefield fundus fluorescein angiography (FFA) with the use of Optos 200Tx (Optos plc, Dunfermline, United Kingdom), and B-ultrasound, if required. BCVA was recorded and then transformed into the logarithm of the minimal angle of resolution (logMAR) scale to carry out an investigation on the statistical level. The counting fingers vision value was 0.01 (2.0 logMAR) and the hand movement value was determined to be 0.001 (3.0 logMAR). was examined by built-in measurement tools in the same machine with the use of its tracking software (i.e., FastTrac™ retinal-tracking technology), in terms of the respective follow-up visit. The cases were examined at baseline, 1 week, 1 month, and 3 months after the completion of the surgery.

### Intervention

All patients underwent a standard 25-gauge PPV based on the 25-gauge constellation system (Alcon, Fort Worth, TX, United States) under local anesthesia. A speed of 5,000 cuts per minute was used for vitrectomy, including core vitrectomy and posterior hyaloid removal. Cataract phacoemulsification combined with intracapsular intraocular lens implantation was carried out if the study eye was phakic. After PVD was induced, ERM was peeled with the use of intraocular forceps, and then, 3 × 3 papilla diameter (PD) internal limiting membrane (ILM) was peeled with the assistance of indocyanine green (ICG) under a high-magnification viewing system. Patients in the DEX group received a 0.7 mg Ozurdex (Allergan, Inc., Irvine, CA) intravitreal implant at the end of the surgery and were recommended to avoid the face-down position to prevent Ozurdex dislocation into the anterior chamber. The same experienced vitreoretinal specialist (QJF) carried out all surgical procedures. All patients received antibiotics four times per day for 14 days. The DEX group was subjected to topical non-steroidal drugs with the capability of inflammation reduction (bromfenac) four times per day for 4 weeks, and the non-DEX group employed 1% prednisolone eye drops four times a day for 7 days with a tapered frequency in the 4 weeks after the completion of the surgery.

### Statistics

The baseline characteristics of all patients were collected and analyzed using the SPSS Statistics 19.0 software (IBM SPSS Inc., Chicago, United States). Normally distributed continuous variables are presented as the mean ± standard deviation (SD). Non-normally distributed continuous variables are presented as the median (interquartile range (IQR)). Student’s *t*-test was used to compare normally distributed quantitative variables, while the non-parametric Wilcoxon signed-rank test was used for non-normally distributed quantitative variables. Categorical data were analyzed using Pearson’s χ^2^ test or Fisher’s exact test. A threshold of *p*-value <0.05 was set for statistical significance.

## Results

### Baseline demographics

**FIGURE 1:**
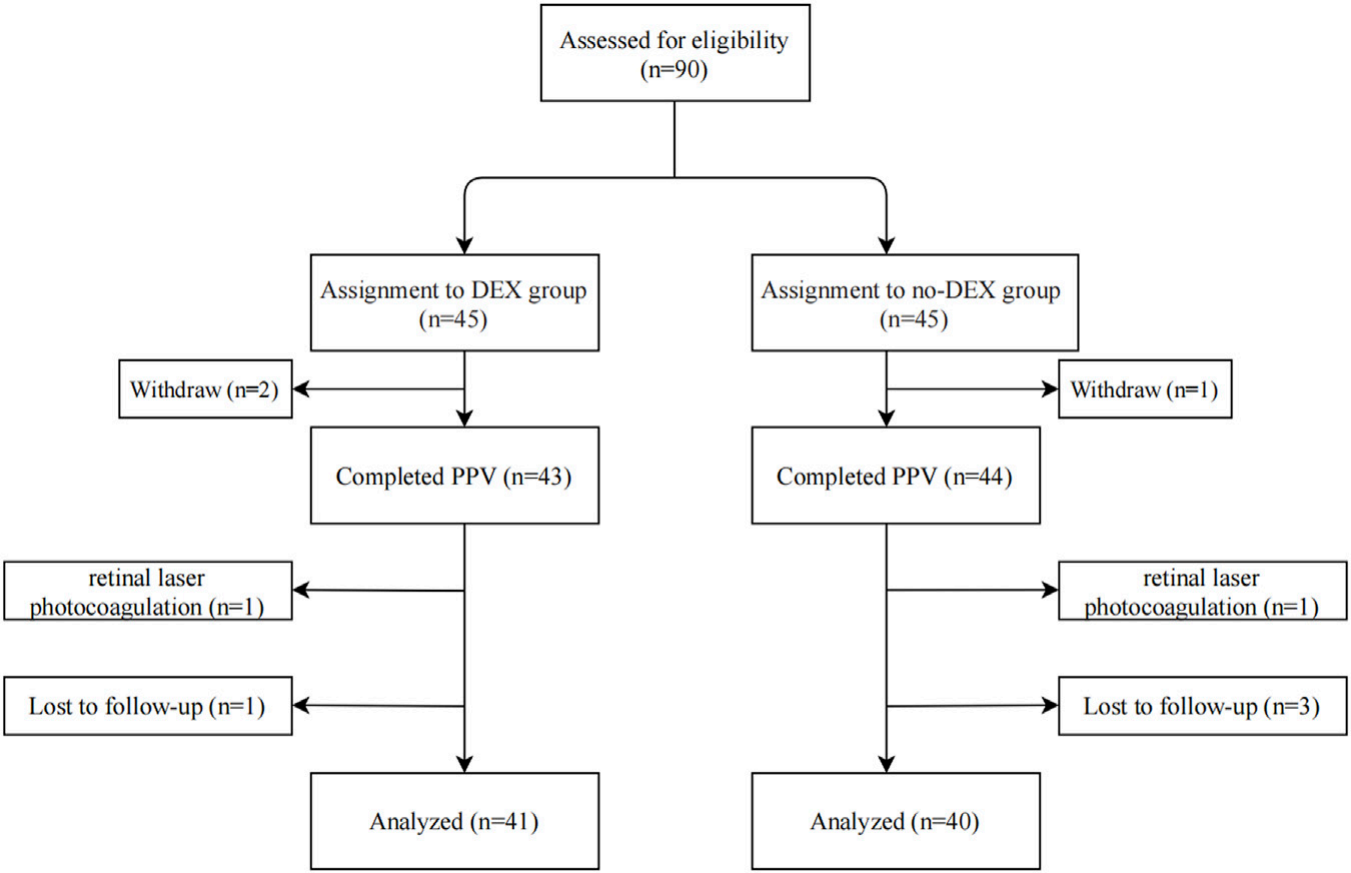
Flowchart indicating the study population’s distribution. DEX, dexamethasone; PPV, pars plana vitrectomy.

In all, 90 eyes of 90 cases were recruited and were divided into two groups in a random manner, i.e., 45 eyes of 45 cases in the DEX group and 45 cases in the non-DEX group. Three eyes were withdrawn (two in the DEX group and one in the non-DEX group) before PPV. Two eyes (one each in the DEX and non-DEX groups) were removed from the protocol for retinal laser photocoagulation used during surgery, and four eyes were lost to follow-up (one in the DEX group and three in the non-DEX group) in the 3-month follow-up period. Finally, 41 eyes of 41 patients in the DEX group and 40 eyes of 40 patients in the non-DEX group were included and analyzed in this study (Figure 1). The baseline characteristics and demographics of all included patients in both groups are listed in Table 1. There were no statistically significant differences in the age, sex, duration of the disease, body mass index (BMI), DM, hypertension, status of lens, baseline disorganization of retinal inner layers (DRILs), ellipsoid zone (EZ)/external limiting membrane (ELM) disruption, ectopic inner foveal layers (EIFLs), CRT, BCVA, and IOP between the two groups (Table 1).

**TABLE 1:** Patient characteristics.

Characteristic	DEX group (*n* = 41)	Non-DEX group (*n* = 40)	*p*-value
Eyes/patients	41/41	40/40	-
Age (years)	63.50 ± 7.13	67.28 ± 8.67	0.522
Male/female	22/19	17/23	0.315
Duration (m)	15.51 ± 4.33	17.47 ± 6.34	0.685
BMI (kg/m^2^)	20.57 ± 2.13	22.01 ± 1.99	0.477
DM, *n* (%)	10 (24.4)	7 (17.5)	0.446
Hypertension, *n* (%)	9 (22.0)	11 (27.5)	0.563
Pseudophakic, *n* (%)	7 (17.1)	12 (30)	0.170
DRIL, *n* (%)	1 (2.4)	2 (5.0)	0.542
ELM/EZ disruption, *n* (%)	2 (4.9)	3 (7.5)	0.624
EIFL thickness (mm)	154.22 ± 70.16	179.68 ± 68.21	0.214
EIFL stage, *n* (%)	1 (2.4)	1 (2.5)	0.984
Stage 1			
Stage 2	22 (53.7)	21 (52.5)	
Stage 3	13 (31.7)	14 (35)	
Stage 4	5 (12.2)	4 (10)	
CRT (mm)	423.99 ± 89.98	404.61 ± 98.81	0.376
BCVA (logMAR)	0.62 ± 0.57	0.67 ± 0.63	0.499
IOP (mmHg)	15.51 ± 3.70	14.77 ± 3.55	0.556

DEX, dexamethasone; BMI, body mass index; DM, diabetes mellitus; DRIL, disorganization of the retinal inner layer; ELM, external limiting membrane; EZ, ellipsoid zone; EIFL, ectopic inner foveal layer; CRT, central retinal thickness; BCVA, best-corrected visual acuity; IOP, intraocular pressure.

### Outcomes

CRT in the DEX group was lower than that in the non-DEX group at 1 month postoperatively (*p* <0.05), whereas no significant differences were reported at 1-week and 3-month follow-up (*p* = 0.109 and *p* = 0.417, respectively) (Table 2).

**TABLE 2:** Comparison of CRT at each visit.

CRT (mm)		DEX group (*n* = 41)		Non-DEX group (*n* = 40)	*p*-value
Baseline		423.99 ± 89.98		404.61 ± 98.81	0.376
Postoperative 1 week		361.24 ± 67.33		389.90 ± 80.61	0.109
Postoperative 1 month		253.96 ± 70.52		303.14 ± 69.55	0.023*
Postoperative 3 months		233.95 ± 0.58		247.99 ± 0.48	0.417

CRT, central retinal thickness; DEX, dexamethasone.

Nevertheless, postoperative BCVA did not change in sync with CRT. BCVA in both groups was improved during the follow-up (from 0.66 ± 0.63 to 0.39 ± 0.48 logMAR in the non-DEX group and from 0.62 ± 0.57 to 0.35 ± 0.58 logMAR within the DEX group). Although the most significant difference of logMAR BCVA was identified in the first month of the follow-up visit, there were no statistical differences with respect to BCVA between the two groups at any point of the follow-up visit period (*p* = 0.499, 0.309, 0.246, and 0.517, respectively) (Table 3). The IOP in each group increased at an early phase; however, it tended to decline even though no statistical differences were reported in the two groups at the respective visit points (*p* = 0.556, 0.639, 0.741, and 0.517, separately) (Table 4).

**TABLE 3:** Comparison of BCVA at each visit.

BCVA (logMAR)		DEX group (*n* = 41)		Non-DEX group (*n* = 40)	*p*-value
Baseline		0.62 ± 0.57		0.66 ± 0.63	0.499
Postoperative 1 week		0.47 ± 0.63		0.52 ± 0.61	0.309
Postoperative 1 month		0.39 ± 0.52		0.45 ± 0.55	0.246
Postoperative 3 months		0.35 ± 0.58		0.39 ± 0.48	0.517

BCVA, best-corrected visual acuity; DEX, dexamethasone.

**TABLE 4:** Comparison of IOP at each visit.

IOP (mmHg)		DEX group (*n* = 41)		Non-DEX group (*n* = 40)	*p*-value
Baseline		15.51 ± 3.70		14.77 ± 3.55	0.555
Postoperative 1 week		18.24 ± 5.33		12.90 ± 6.61	0.109
Postoperative 1 month		17.96 ± 7.52		13.14 ± 6.55	0.341
Postoperative 3 months		14.95 ± 3.58		13.99 ± 3.48	0.517

IOP, intraocular pressure; DEX, dexamethasone.

**FIGURE 2:**
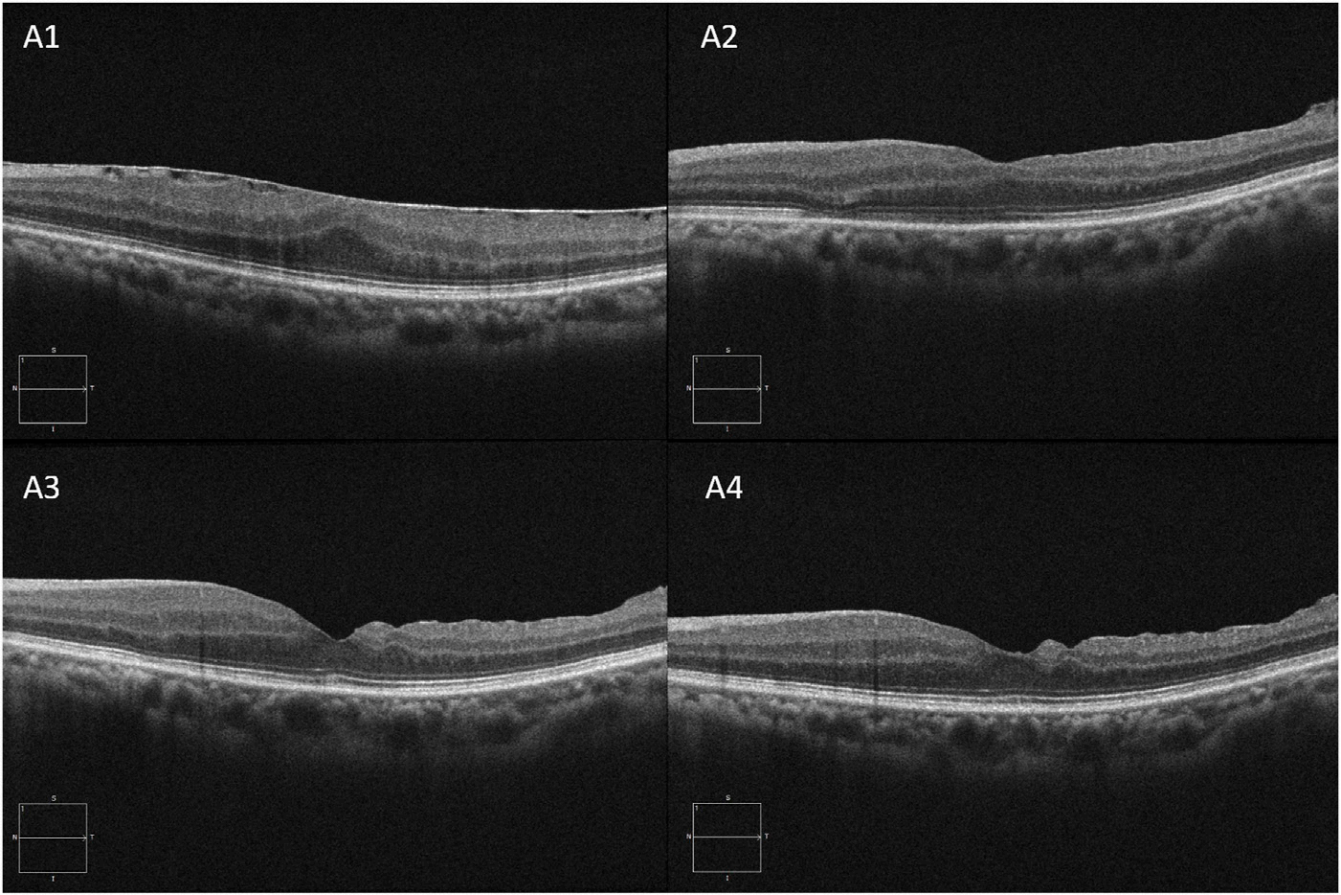
A 58-year-old woman complained of distorted vision for 8 months; she underwent 25-gauge (G) PPV combined with ERM and ILM peeling surgery and DEX implant surgery. **A1, A2, A3**, and **A4** are OCT images at baseline, 1 week, 1 month, and 3 months of the postoperative visit, respectively.

**FIGURE 3:**
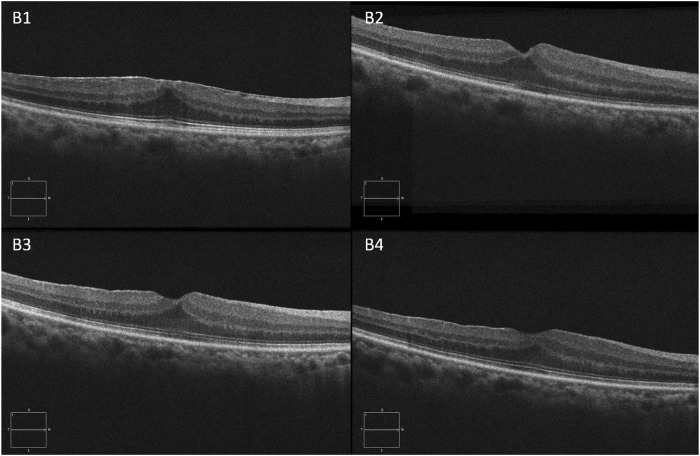
A 69-year-old man complained of visual acuity loss with visual distortion for 1 year. The patient underwent 25-G PPV combined with ERM and ILM peeling surgery. **B1, B2, B3,** and **B4** are OCT images at baseline, 1 week, 1 month, and 3 months of the postoperative visit, respectively.

The illustrations of each follow-up visit of the two groups after surgery are shown in Figure 2 and Figure 3.

## Discussion

PPV with membrane peeling has been confirmed as the gold standard of treating patients with symptomatic ERMs, and the majority of cases achieve a favorable outcome, along with visual acuity enhancement (Mitsui et al., 2016; Mahmoudzadeh et al., 2022). Nevertheless, it usually takes several months to up to 1 year for visual recovery with the slow normalization of the retinal morphology (Kim et al., 2010). Furthermore, persistent residual intraretinal edema can still present sometimes, and a complete visual function recovery is less likely to be achieved. The mechanism of macular edema in idiopathic ERM remains unclear. As revealed by the results of Katira et al. (2008) and Ahn et al. (2012), it shows a correlation with the blood–retinal barrier breakdown, which arose from the release of inflammation-related cytokines, the growth factors of preoperative mechanical traction after the completion of the operation and intraoperative manipulation. As indicated by Mandelcorn et al. (2003), immunostaining for transforming growth factor-beta (TGF-beta 2) and the vascular endothelial growth factor (VEGF) was positive within 85% of idiopathic ERM samples, thus suggesting that underlying chronic inflammation can be present in idiopathic ERM.

Corticosteroids are very potent anti-inflammatory agents, capable of blocking several pathological processes and playing a certain role in macular edema development in several ways: the inhibition of VEGF synthesis, prostaglandins, and a considerable number of proinflammatory cytokines; stabilization of endothelial cell tight junctions; leukocyte migration prevention; and the reduction of fibrin deposition (Chang-Lin et al., 2011). Nevertheless, the administrating route and corticosteroid categories are likely to exert a certain effect on the efficacy in specific diseases. Systemic corticosteroids may result in certain adverse events, such as adrenal suppression, Cushingoid state, osteoporosis, and exacerbation of diabetes (Whittier and Saag, 2016). Local or topical administration generally results in a concentration of the drug that is suboptimal within the vitreous (Weijtens et al., 2002). Accordingly, the direct intravitreally injecting process of corticosteroids may serve as a more effective way of achieving a concentration of the drug that is optimal within the vitreous. In comparison with TA, dexamethasone is soluble in water and exhibits the least possibility of aggregating within the trabecular meshwork to result in an IOP increase. TA is cleared more rapidly in vitrectomized eyes, while DEX exhibits a consistent pharmacokinetic profile in both non-vitrectomized and vitrectomized eyes. The development of DEX can lead to the optimization of drug delivery control, while the rate of adverse events may decline and lead to a reduction in frequent intraocular injections in vasectomized eyes (Furino et al., 2014; Hattenbach et al., 2017). DEX has been adopted to contribute to the elimination of persistent retinal edema after epiretinal membrane peeling with a mean percentage reduction of 25% in central foveal thickness (Taney et al., 2015). In this study, we identified that CRT decreased more rapidly in the DEX group than in the non-DEX group at the 1-month follow-up visit, consistent with the results of other relevant studies (Iovino et al., 2019; Sane et al., 2020). Along with the clearance of DEX, the reduction of CRT slowed down in the DEX group, which may explain the result of the comparable CRTs of the two groups at the 3-month visit.

Although a significant CRT reduction at 1 month after the operation was observed in the two groups, we failed to find a similar improvement in BCVA. A systematic review by Scheerlinck et al. (2015) revealed that CRT was not associated with postoperative BCVA. In fact, postoperative BCVA may be affected by multiple factors. One main factor is the integrity of outer retinas at baseline in eyes with ERMs (Shimozono et al., 2012). Some research studies have suggested that there is a correlation between vision loss and internal retinal damage. Zur et al. highlighted that the DRIL can also be used to estimate BCVA after the operation (Zur et al., 2018). Some investigators reported that dissociated optic nerve fiber layer, which was correlated with ILM peeling, could also cause postoperative central scotoma and worse BCVAs (Pan et al., 2014). Furthermore, some research studies have suggested that EIFL, as defined by the presence of continuous hyper-reflective and hypo-reflective bands that extend based on the internal nuclear layer and internal plexiform layer across the foveal region, showed a negative correlation with BCVA improvement after an operation in ERM cases (Doguizi et al., 2018; Govetto et al., 2019; González-Saldivar et al., 2020). However, there were also investigations that reported no associations between them. The percentage of eyes with DRIL, EIFL, or ELM/EZ band disruptions at baseline was comparable between the two groups in our study. So, these three possible reasons seemed to be unable to explain the discrepancy of CRT and BCVA variations at 1 month in our study. The factors correlated with the BCVA after the operation of PPV for ERM cases still need further *post hoc* investigations.

A systematic review and meta-analysis showed that intravitreal dexamethasone implants can improve BCVA and CRT in macular edema eyes secondary to vitrectomy for ERM and retinal detachment (Parisi et al., 2021). Macular edema after vitrectomy seems to be closely related to inflammation. Dexamethasone can greatly reduce the inflammatory response. In our study, we did not find a significant advantage in our DEX group. It may be related to the variable incidence of postoperative macular edema and the fact that we had peeled ILM, which can accelerate the resolution of macular edema to some extent. Based on relevant research studies and our results, we hypothesized that eyes with higher preoperative foveal thicknesses or severe postoperative macular edema might benefit more through PPV combined with Ozurdex.

The safety and efficacy of 0.7 mg Ozurdex in vitrectomized eyes have been proved in several studies. The most frequent ophthalmic adverse effect is the transient elevation of IOP (Boyer et al., 2011; Medeiros et al., 2014). There were five patients (12%) in the DEX group experiencing IOP elevations of 10 mmHg or more from baseline at 1 month, postoperatively, in our study, whereas it was well controlled by single eye drops for lowering IOP. No cases at 3 months of follow-up required novel IOP-reducing medications. During the follow-up, we did not report any differences that achieved statistical significance in the variation of IOP between the two groups.

There are several limitations to our study. First, being a single-center study with a relatively small sample size, the generalizability of our conclusion has yet to be confirmed. Second, this was a study carried out in Chinese patients only; whether or not similar results can be found in other ethnicities still needs further investigation. Third, data from more surgeons could have been included; however, inter-surgeon variability in patient selection and postoperative management may have influenced the accurate interpretation of the results. Finally, we did not analyze the OCT imaging findings of individual retinal layers, which may or may not have shown differences between the DEX and non-DEX groups, although this was not the aim of this study. Future evaluations may address such issues.

In conclusion, a combined treatment with vitrectomy and intravitreal corticosteroid injections may accelerate CRT reduction at an early stage after ERM surgery. However, it did not result in a significant change in CRT and BCVA compared with vitrectomy alone at the end of follow-up.

## Data Availability

The original contributions presented in the study are included in the article/Supplementary Material; further inquiries can be directed to the corresponding author.

## Abbreviations

DEX, dexamethasone; BCVA, best-corrected visual acuity; ERM, epiretinal membrane; PPV, pars plana vitrectomy; ILM, internal limiting membrane; CRT, central retinal thickness; IOP, intraocular pressure; TA, triamcinolone acetonide; DM, diabetes mellitus; SD-OCT, spectral-domain optical coherence tomography; FFA, fundus fluorescein angiography; logMAR, logarithm of the minimal angle of resolution; ILM, internal limiting membrane; ICG, indocyanine green; IQR, interquartile range; BMI, body mass index; DRIL, disorganization of the retinal inner layer; EZ, ellipsoid zone; ELM, external limiting membrane; EIFL, ectopic inner foveal layer; TGF-beta 2, transforming growth factor-beta; and VEGF, vascular endothelial growth factor.
